# Developing clinically practical transcranial direct current stimulation protocols to improve saccadic eye movement control

**DOI:** 10.16910/jemr.10.3.5

**Published:** 2017-06-05

**Authors:** Po Ling Chen, Liana Machado

**Affiliations:** Department of Psychology and Brain Health Research Centre, University of Otago and Brain Research, New Zealand; Department of Psychology and Brain Health Research Centre, University of Otago and Brain Research, New Zealand

**Keywords:** Electrical brain stimulation, eye movement, eye tracking, saccades, antisaccades, oculomotor control

## Abstract

Recent research indicates that anodal transcranial direct current stimulation (tDCS) applied over the frontal eye field (FEF) can improve saccadic eye movement control in healthy young adults. The current research set out to determine whether similar results can be produced using a clinically practical protocol, whether tDCS applied over the dorsolateral prefrontal cortex (DLPFC) might also afford benefits, and whether benefits extend to older adults. Twenty young and 10 older adults completed two active (FEF and DLPFC) and one sham stimulation session. To aid clinical translation, the method of positioning the electrodes entailed simple measurements only. Saccadic performance following anodal tDCS applied over the FEF or DLPFC did not differ from the sham condition in either age group. Additionally, saccadic performance contralateral to the active electrodes showed no evidence of benefits over ipsilateral performance. These results call into question whether the protocol utilized can be applied effectively using only simple measurements to localize the relevant frontal subregion. Future efforts to develop a clinically practical tDCS protocol to improve saccadic eye movement control should include a sham control condition and consider adjusting the tDCS electrode montage and current strength to optimize the chances of conferring benefits in the population under study.

## Introduction

 Over the past 10 years, transcranial direct current stimulation (tDCS) has been widely used to modulate cortical excitability to the benefit of cognitive and motor functions, in both healthy and clinical populations ( 1 ). However, studies investigating the effects of tDCS on control over eye movements have been scarce. Only recently evidence has emerged demonstrating that positively charged anodal tDCS applied over the frontal eye field (FEF) can be used to improve saccadic eye movement control in healthy young adults ( 2 ). Given that healthy aging and a large number of age-related clinical conditions (e.g., mild cognitive impairment and Alzheimer’s and Parkinson’s disease) are associated with reduced control over the eye movement system, particularly when a high level of strategic control is required ( 36 ), findings in Kanai, Muggleton ( 2 ) are exciting as they suggest that anodal tDCS may be a useful therapeutic tool for improving voluntary control over the oculomotor system in impaired populations. 

 Voluntary control over saccadic eye movements involves complex underlying neuromechanisms by which cortical oculomotor regions must be able to impose topdown regulation over subcortical oculomotor regions ( 7 ). The antisaccade paradigm ( 8 ) is a tool commonly used for behavioral measurement of voluntary control over saccadic eye movements. A successful antisaccade involves moving the eyes in the opposite direction when a stimulus suddenly appears in the peripheral visual field. This capability involves two control processes: 1) suppressing an unwanted reflexive prosaccade toward the peripheral stimulus; 2) voluntarily generating an eye movement away from the peripheral stimulus to the mirror position ( 9, 10 ). The frontal subregions most commonly posited to underpin accurate performance of antisaccades are the FEF and dorsolateral prefrontal cortex (DLPFC; 11, 12). However, as reviewed in Chen and Machado ( 13 ), the contributions of the FEF and DLPFC to the suppression of reflexive prosaccades and the generation of correct antisaccades remain unclear. While the literature is in agreement that the FEF is the key region supporting generation of volitional eye movements, there is disagreement as to which frontal subregion supports suppression of reflexive eye movements. Specifically, some have reported evidence implicating the FEF as the key region supporting suppression of reflexive eye movements ( 14 ), while others have claimed that DLPFC (Brodmann’s area 46) plays the main role in suppressing reflexive eye movements, as reviewed in PierrotDeseilligny, Milea ( 15 ). 

 The one study ( 2 ) that assessed the influences of tDCS over cerebral cortex on oculomotor behavior found that in healthy young adults anodal tDCS over the FEF influenced subsequent antisaccade performance such that reflexive errors were reduced contralaterally without any effect on correct antisaccade latencies, and in addition subsequent correct prosaccade latencies were shortened contralaterally. These findings indicate that while anodal tDCS over the FEF facilitates suppression of unwanted contraversive reflexive eye movements, it also speeds the latencies of wanted contraversive reflexive eye movements. These anodal tDCS benefits peaked 10 to 30 minutes post stimulation. In this seminal study, electrode positioning over the FEF was determined based on predefined standardized coordinates using structural magnetic resonance imaging (MRI) of each individual. In the current study, we tested whether the benefits reported in Kanai, Muggleton ( 2 ) can be induced using a more clinically practical protocol that does not entail expensive tools or time consuming procedures (e.g., MRI) to determine electrode positioning, as using such tools falls outside available resources in many clinical settings. 

 In addition to assessing anodal tDCS over the FEF, in the current study we assessed whether applying anodal tDCS over DLPFC might also benefit saccadic eye movement control, especially with respect to suppressing unwanted reflexive saccades, as one might predict based on human brain lesion studies ( 16-18 ). Furthermore, in the current study, we assessed whether saccadic eye movement control benefits extend to older adults. Ample evidence from non-oculomotor studies indicates tDCS confers more robust cognitive benefits in older adults ( 19 ), presumably due to far more room for improvement and thus greater potential for benefit. However, no studies to date have assessed whether the same applies to saccadic eye movement control. In testing the efficacy of tDCS to improve saccadic eye movement control, we compared oculomotor behavior ipsilateral versus contralateral to the anodal electrode (as per 2) and we also included a sham control condition (in contrast to 2). This enabled us to determine whether performance contralateral to the FEF and DLPFC electrodes was superior to ipsilateral performance, and also whether it was superior to performance contralateral to sham stimulation. 

In sum, the purpose of the current study was threefold: 1) determine whether benefits of anodal tDCS on saccadic eye movement behavior can be induced using a clinically practical protocol; 2) determine whether anodal tDCS over DLPFC also benefits saccadic eye movement control; 3) determine whether benefits extend to older adults.

## Methods

### Participants

 Thirty adult males, 20 young (age range = 20-25 years, mean = 22.2, *SD* = 1.0; education range = 15-18 years, mean = 16.2, *SD* = 1.2) and 10 older (age range = 65-70 years, mean = 68.6, *SD* = 1.1; education range = 10-31 years, mean = 15.1, *SD* = 5.4) from the Dunedin community, New Zealand, participated and were reimbursed NZ$15 per session. Participants were all righthanded according to the Measurement of Handedness ( 20 ). All participants reported having normal or corrected vision; no pace maker, implanted electronic device or metal implants; no history of, and not currently taking any medications for neurological or psychiatric problems; no chronic skin conditions; and abstained from recreational drugs and alcohol in excess of three units during the 24 hours prior to their testing session. Participants also completed a depression inventory the Center for Epidemiologic Studies Depression Scale (CES-D; 21), which has a maximum score of 60. Of the young adults, 11 scored below 16, indicating they had no clinical symptoms of depression and nine scored between 16 and 22, indicating subthreshold depression symptoms. Of the older adults, five scored below 16, indicating they had no clinical symptoms of depression and five scored between 16 and 21, indicating subthreshold depression symptoms. Older adults were also screened for dementia using the Mini-Mental State Examination (MMSE; 22); all scored at least 26 out of 30, which indicates none were demented. This study was approved by the University of Otago Human Ethics Committee (H13/123) and was performed in accordance with the relevant guidelines and regulations. All participants gave informed consent prior to participation.

### Design

 The current study employed a randomized, singleblind, sham-controlled, crossover experimental design. As per Kanai, Muggleton ( 2 ), in the current study half of the participants in each age group were randomly assigned to have the anodal electrode positioned over the left hemisphere and half over the right. All participants completed three sessions of stimulation: active over each frontal subregion (FEF and DLPFC) and sham over an intermediate frontal subregion, with the order of the stimulation conditions counterbalanced across participants within each age group, and each session separated by a minimum of 7 days. Each session lasted about 1 hr. 

### Electrodes Positioning

 The 10-20 system for electroencephalography (EEG; 23) was used to determine the placement of the anodal electrode over the assigned hemisphere. The anodal electrode was positioned for the FEF condition 1.5 cm anterior and 20% laterally from the vertex ( 24, 25 ), for the DLPFC condition 5 cm anterior and 20% laterally from the vertex ( 26, 27 ), and for the sham condition 2.5 cm anterior and 20% laterally from the vertex (between the FEF and DLPFC positions). In all cases, the reference electrode (cathode) was positioned on the upper arm (just below the shoulder) ipsilateral to anodal electrode. Prior to proceeding, inspection of all sites of stimulation confirmed there were no lesions or signs of skin irritation. 

### tDCS Protocol

 A constant current 9 volt battery driven device (ActivaDose II) delivered 1 mA direct current through carbon rubber electrodes placed in sponge pockets soaked in saline solution. As per Kanai, Muggleton ( 2 ), the anodal electrode, which was 3 x 3 cm, delivered a current density of 0.11 mA/cm² , and the reference electrode, which was 5 x 7 cm, delivered a current density of 0.03 mA/cm² . The intensity of the current slowly ramped up to 1 mA over the initial 10 s of stimulation. During active stimulation current was delivered for 10 minutes, and during sham stimulation the device was turned off 30 s after the start of stimulation. At the end of each stimulation period, participants completed a questionnaire designed to monitor adverse effects. No adverse effects were reported, as might be expected given that the current density was 50 times lower than the previously studied safety threshold ( 28 ), and was also lower than the current densities used in many studies that were well tolerated and considered to be safe ( 29 ). When questioned at the end of their final session, no participants could differentiate between the active and sham conditions. 

### Eye Movement Testing

**Figure 1:**
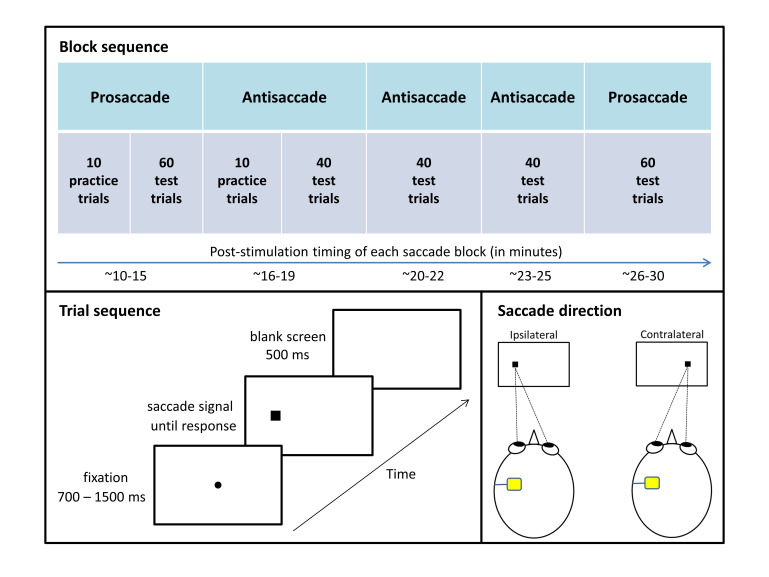
Figure 1. Eye movement testing protocol. Each session entailed completion of five eye movement blocks, which differed only in the required response: look at the peripheral stimulus during prosaccade blocks and look at the mirror opposite position during antisaccade blocks. Responses were coded relative to the position of the anodal electrode (saccade directed ipsilaterally or contralaterally).

Figure 1 summarizes the eye movement testing protocol, which was adapted from Antoniades, Ettinger ( 30 ). In order to target the post-stimulation time period that showed anodal tDCS benefits in Kanai, Muggleton ( 2 ), in the current study eye movement testing commenced 10 minutes post stimulation. Participants completed five eye movement blocks in this order: one block of prosaccades, three blocks of antisaccades, and then a second block of prosaccades. Between blocks, participants were provided with a 1 minute break. Between blocks of different types, the experimenter informed participants of the type of saccades required and instructed them in how to respond. Participants wore a head-mounted eye tracker (Model 310, Applied Science Laboratories, Massachusetts, USA) and sat 57 cm away from a computer screen in a dimly lit room, with distance maintained via a chinrest. The experimenter calibrated the eye-tracking system before each block. Stimuli were presented on a white background via MATLAB (The MathWorks, Natick, MA) and The Psychophysics Toolbox ( 31, 32 ).

For prosaccade and antisaccade blocks, each trial commenced with the appearance of a black fixation dot extending 0.3° of visual angle and centered on the screen. After a variable interval (700, 900, 1100, 1300, or 1500 ms), the fixation dot disappeared and a black square subtending 1° appeared 8.5° to the left or right of center (measured to the center of the square). Fixation dot offset and peripheral square onset occurred simultaneously. Participants were instructed to respond to the appearance of the square as quickly as they could without compromising accuracy by looking at it during prosaccade blocks and by looking in the opposite direction during antisaccade blocks. During practice trials, a 900 Hz error tone sounded for 300 ms if participants made no response, responded in the wrong direction, or responded in less than 50 ms or more than 1000 ms after saccade signal onset (i.e., the appearance of the peripheral square). The screen went blank for 500 ms between trials. Saccade signal position (left or right) and fixation duration (700, 900, 1100, 1300, or 1500 ms) were randomly selected for each trial with the constraint that each combination of conditions was equally likely to occur across the test trials. Each prosaccade test block had 60 trials and each antisaccade test block had 40 trials, and participants were given 10 practice trials at the beginning of the first block of each saccade type. Practice trials were repeated upon request by participants or if the experimenter identified the participant did not understand the instruction.

Horizontal position of the right eye was sampled at 1100 Hz. When the right eye exceeded the horizontal velocity of 50°/s with at least 1° amplitude, the movement was defined as a saccade. The program then recorded the latency of saccade onset (by backtracking until the velocity dropped below 10°/s) and the direction of movement. During the trials, the experimenter manually rejected responses contaminated by blinking or other factors such as sneezing or coughing. In addition, trials were excluded from analysis if eye position at the time of saccade signal onset deviated from center by more than 3°, or if the latency was shorter than 50 ms or longer than 1000 ms.

### Statistical Analyses

 For each participant, the measured variables of interest were correct median reaction times (RTs) and percentage of reflexive errors during antisaccade blocks as a function of stimulation condition (FEF, DLPFC, or sham) and saccade direction (ipsilateral or contralateral to the anodal electrode). Shapiro-Wilk test was used to determine the normality of each data set. When assumptions of parametric tests were violated, non-parametric tests confirmed the parametric results. In cases where sphericity was violated (*p*( < .05), a Greenhouse-Geisser correction was applied when Epsilon ranged from .70 to .90, otherwise a multivariate test (Pilai’s Trace) was applied. The alpha level was *p* < .05. The sample size was chosen based on Kanai, Muggleton ( 2 ), which reported significant results for contralateral versus ipsilateral performance in a group of 16 young adults; a power analysis computed using G*Power 3.1.9.2 ( 33 ) indicated our study had 87% power to detect a similar effect size (*d*_z _ = 0.5875), and thus beta was 0.13. Note that stimulation was always applied unilaterally, and the results were coded based on whether the saccade was directed ipsilaterally or contralaterally to the stimulated hemisphere (see Figure 1, lower right panel, for examples). 

## Results

 To determine if performance varied across the blocks, initial repeated-measures analyses of variance (ANOVAs), with stimulation condition, saccade direction, and saccade block as factors, were performed for each of the measured variables of interest (prosaccade latencies, antisaccade latencies, and reflexive error rates during antisaccades). The results revealed no main effect of block for the latency variables, but there was a main effect of block for reflexive error rates during in responsiveness to brain stimulation ( 34 ), to determine whether a subset of the participants benefitted from active stimulation, each individual’s data was checked for any apparent asymmetries in the active stimulation conditions consistent with superior performance contralateral versus ipsilateral, and if so contralateral active versus sham; paired-samples *t* tests tested whether any of the differences reached significance. Table 1 summarizes the mean of the median correct response latencies and reflexive error rates during antisaccades for each stimulation condition in each age group. Tables S1 and antisaccades, *F*(2, 58) = 9.398, * p* < .001, *r* = .495, reflecting increasing reflexive error rates across blocks, presumably due to fatigue. However, since saccade block did not interact with stimulation condition or saccade direction for any of the measured variables of interest (all *p*s > .200), the data were collapsed across blocks in the mixed ANOVAs reported below, all of which included age group as a between-participant factor, and stimulation condition and saccade direction as within-participant factors. Regardless of the ANOVA results, paired samples *t* tests assessed hemispheric asymmetries in the active conditions and differences against sham stimulation. In addition, in light of individual differences S2 (in Appendix) detail the results of each group-level *t* test. 

**Table 1:**
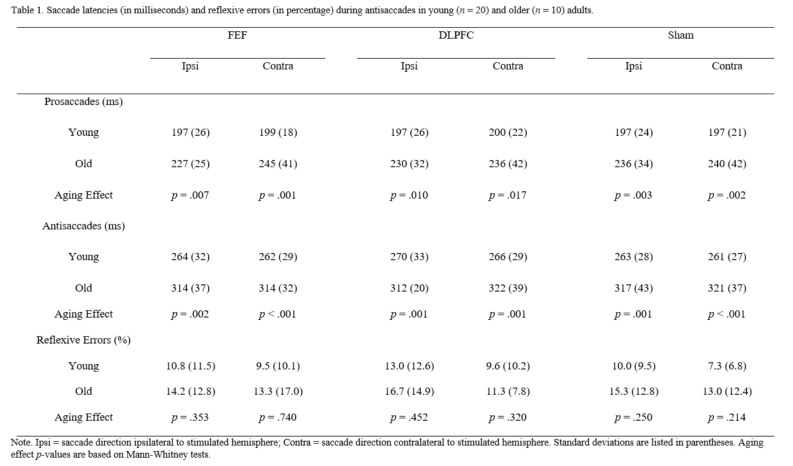
Table 1. Saccade latencies (in milliseconds) and reflexive errors (in percentage) during antisaccades in young (*n*=20) and older (*n*=10) adults.

### Prosaccade Latencies

**Figure 2:**
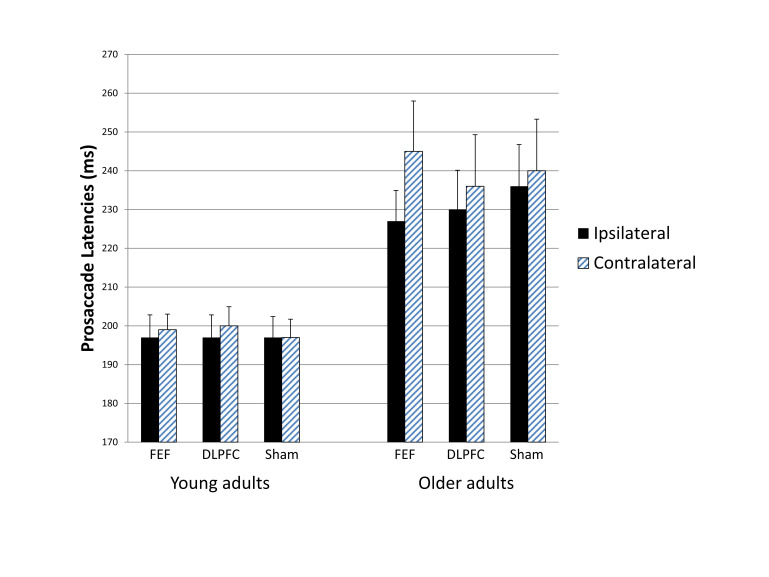
Figure 2. Prosaccade latencies ipsilateral versus contralateral to the stimulated hemisphere for each stimulation condition in each age group. Neither of the active stimulation conditions shortened latencies contralaterally relative to ipsilaterally. Bars indicate standard errors.

Figure 2 summarizes the latency data for the prosaccade blocks. The mixed ANOVA revealed a significant main effect of age group, *F*(1, 28) = 16.102, *p* < .001, *r* = .604, reflecting longer latencies in older than young adults. The expected two-way interaction between stimulation condition and saccade direction approached significance, * F*(2, 56) = 2.455, *p* = .095, *r* = .285; however, in contrast to the expected shortening of contralateral relative to ipsilateral prosaccade latencies, contralateral latencies tended to be longer particularly in the FEF stimulation condition, although a paired-samples *t* test showed that this trend for an asymmetry in the FEF stimulation condition did not reach significance (*p*( = .159). The three way interaction did not approach significance, *F*(2, 56) = 1.684, *p* = .195, *r* = .239, and as can be seen in Figure 2 neither age group exhibited the predicted pattern of faster contralateral prosaccades. No other main effects or interactions approached significance (all *p*s > .100). Pairedsamples *t* tests computed for the full age-mixed sample confirmed no ipsilateral versus contralateral latency differences in either active stimulation condition (FEF or DLPFC) and no differences relative to the sham stimulation condition (all *p*s > .100; see Table S1 for details). Similarly, *t* tests computed for each age group confirmed no ipsilateral versus contralateral latency differences in either active stimulation condition and no differences relative to the sham stimulation condition (all *p*s > .100; see Table S2F for details). 

 Consideration of each individual’s data also indicated a lack of benefits. In the FEF stimulation condition, only three of the 20 young adults and none of the older adults showed significantly faster contralateral relative to ipsilateral latencies, consistent with the pattern reported in Kanai, Muggleton ( 2 ), and only one of these three reached significance when compared with contralateral latencies in the sham stimulation condition, *t*(57) = 2.552, *p* = .013, *Cohen’s d* = 0.676. In the DLPFC stimulation condition, only one of the 20 young adults and none of the older adults showed this asymmetry pattern, and the comparison with the sham stimulation condition did not reach significance (*p*( > .050). 

### Antisaccade Latencies

**Figure 3:**
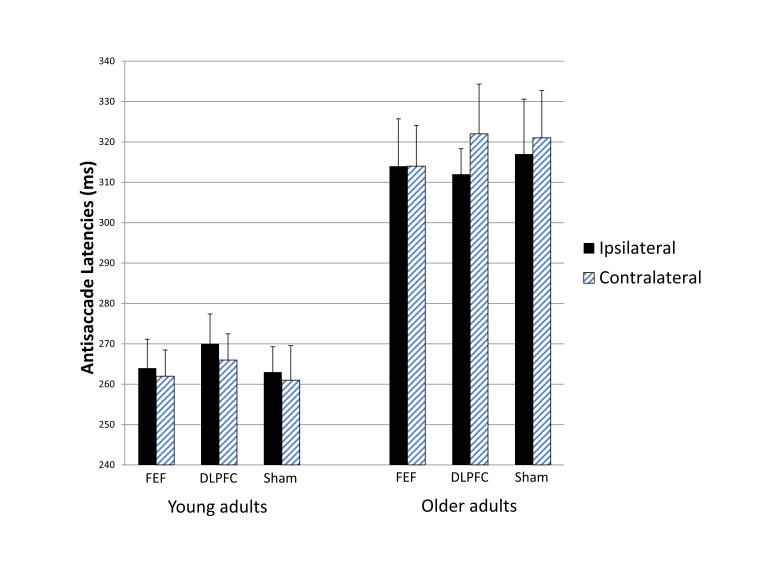
Figure 3. Antisaccade latencies ipsilateral versus contralateral to the stimulated hemisphere for each stimulation condition in each age group. The different stimulation conditions did not differentially influence contralateral versus ipsilateral latencies. Bars indicate standard errors.

Figure 3 summarizes the latency data for the antisaccade blocks. The mixed ANOVA revealed a significant main effect of age group, *F*(1, 28) = 26.643, *p* < .001, *r* = .699, again reflecting longer latencies in older than young adults. Of specific relevance here, the interaction between stimulation condition and saccade direction did not approach significance, *F*(2, 56) = 0.555, *p* = .577, *r* = .138, which indicates that the different stimulation conditions did not differentially influence contralateral versus ipsilateral latencies. Furthermore, stimulation condition and saccade direction did not significantly interact with age group, *F*(2, 56) = 1.905, *p* = .158, *r* = .253. As can be seen in Figure 3, neither age group showed stimulation effects (i.e., asymmetries specific to active stimulation). No other main effects or interactions approached significance (all *p*s > .100). Paired-samples *t* tests confirmed no ipsilateral versus contralateral latency differences in either active stimulation condition (FEF or DLPFC) and no differences relative to the sham stimulation condition (all *p*s > .400; see Table S1 for details). Similarly, t tests computed for each age group confirmed no ipsilateral versus contralateral latency differences in either active stimulation condition and no differences relative to the sham stimulation condition (all *p*s > .100; see Table S2 for details). 

 Consideration of each individual’s data also indicated a lack of benefits. In the FEF stimulation condition, only one of the 20 young adults and one of the 10 older adults showed significantly faster contralateral relative to ipsilateral latencies, and only the young participant reached significance when compared with contralateral latencies in the sham stimulation condition, *t*(38) = 3.017, *p* = .005, *Cohen’s d* = 0.979. In the DLPFC stimulation condition, one of the young adults and none of the older adults showed this asymmetry pattern, and the comparison with the sham stimulation condition did not reach significance (*p*( > .050). 

### Reflexive Error Rates During Antisaccade Blocks

**Figure 4:**
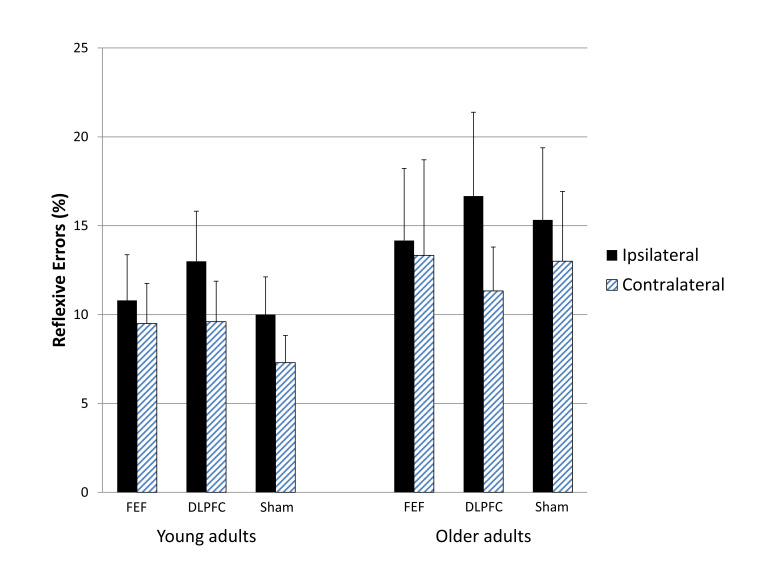
Figure-04. Reflexive error rates toward antisaccade signals positioned ipsilateral or contralateral to the stimulated hemisphere for each stimulation condition in each age group. The same pattern arose for all three stimulation conditions, indicating that neither of the active stimulation conditions was effective at improving suppression of unwanted reflexive prosaccades. Bars indicate standard errors.

Figure 4 summarizes the reflexive error rates during the antisaccade blocks. The mixed ANOVA revealed that, although the data showed the expected trend for higher reflexive error rates in older compared to young adults, the main effect of age group did not approach significance, * F*(1, 28) = 1.252, *p* = .273, *r* = .207, which could be due to the small sample size in the older age group (n = 10). Of specific relevance here, the expected two-way interaction between stimulation condition and saccade direction did not approach significance, *F*(2, 56) = 1.731, *p* = .194, *r* = .241, which indicates that the different stimulation conditions did not differentially influence contralateral versus ipsilateral reflexive errors. In addition, stimulation condition and saccade direction did not interact with age group, * F*(2, 56) = 0.381, *p* = .685, *r* = .114, which suggests that the lack of stimulation effects applies to both age groups. As shown in Figure-04, the pattern of reduced reflexive error rates contralaterally relative to ipsilaterally emerged in all three stimulation conditions, including sham, in both age groups. No other main effects or interactions approached significance (all *p*s > .100). Paired-samples *t* tests confirmed no ipsilateral versus contralateral performance differences in the active FEF stimulation condition and no differences in either active stimulation condition (FEF or DLPFC) relative to the sham stimulation condition (all *p*s > .100); however, fewer reflexive errors were made toward contralateral than ipsilateral saccade signals in the active DLPFC stimulation condition (*p*( = .036; see Table S1 for details), but this asymmetry is unlikely to reflect the tDCS given that performance in the active DLPFC stimulation condition did not differ from performance in the sham condition and moreover that contralateral reflexive error rates were higher in the active DLPFC condition (10.2%) than in the sham condition (9.2%). Separate consideration of each age group showed no ipsilateral versus contralateral latency differences in either active stimulation condition and no differences relative to the sham stimulation condition (all *p*s > .100; see Table S2 for details). 

## Discussion

 Using a more clinically practical protocol, the current study tested whether anodal tDCS over the FEF can induce oculomotor benefits similar to those reported in young adults in Kanai, Muggleton ( 2 ), and in addition assessed whether applying anodal tDCS over DLPFC might also benefit oculomotor behavior and whether these benefits extend to older adults, who are known to have saccadic eye movement control deficits ( 13 ). Overall the results revealed no evidence of oculomotor benefits following anodal tDCS, despite the sample size in the current study exceeding that used in Kanai, Muggleton ( 2 ). Specifically, group analyses showed no differences in the active stimulation conditions relative to sham stimulation, and an asymmetry in saccadic eye movement behavior arose only in the active DLPFC stimulation condition (for reflexive errors in the full mixed-age sample), but this did not reflect better performance relative to sham performance, and the sham condition showed a similar pattern. Analyses of individual participants backed up the null results at the group level, with significant effects relative to sham stimulation occurring in less than 5% of the participants (which is consistent with chance levels, as alpha was set to .05). These results indicate that neither active stimulation site (FEF or DLPFC) afforded better saccadic eye movement control. The absence of oculomotor benefits arose in both age groups, despite the older adults exhibiting the expected saccadic eye movement control deficits that indicate ample room for improvement. These negative outcomes indicate that the clinically practical protocol utilized in the current study was ineffective. 

 One of the main findings reported in Kanai, Muggleton ( 2 ) was that anodal tDCS over the FEF reduced reflexive error rates toward contralateral relative to ipsilateral antisaccade signals. This pattern was also demonstrated in the current study, although the asymmetry did not reach significance. However, as shown in Figure 4, the same pattern also occurred in the sham stimulation condition. Given that Kanai, Muggleton ( 2 ) did not include a sham stimulation condition, it is not possible to determine whether the lower rate of contralateral versus ipsilateral reflexive errors occurred as a result of the tDCS. To determine this, one would need to replicate the protocol used in Kanai, Muggleton ( 2 ) with the addition of a sham stimulation condition. The fact that the current study showed similar asymmetric patterns in the active and sham conditions highlights the need for a sham control comparison condition to confirm whether any observed asymmetries are specifically attributable to tDCS. The other main finding reported in Kanai, Muggleton ( 2 ) is that anodal tDCS over the FEF shortened prosaccade latencies contralateral versus ipsilateral to the stimulated hemisphere. This pattern was not demonstrated in the current study in either age group (see Figure 2). Furthermore, none of the older adults and only three of the 20 young adults showed this pattern, and only one of these three reached significance when compared with sham stimulation, which was not assessed in Kanai, Muggleton ( 2 ). 

 A number of factors could potentially explain the discrepant outcomes. One of the main differences in the design of the current study relative to Kanai, Muggleton ( 2 ) was the lack of precise localization of the FEF. To speed application to better suit clinical environments, in the current study we simplified the tDCS protocol by using basic EEG-based measurements to position the FEF electrode, in accordance with Ro, Cheifet ( 24 ) and Ro, Farne ( 25 ). However, there were several other design differences that may have influenced the results. For example, the saccade paradigm used in the current study (adapted from 30) differed from that used in Kanai, Muggleton ( 2 ), in that in their study permanent boxes marked the possible saccade signal locations (where as the saccade signal locations were unmarked in the current study), the fixation dot overlapped with the saccade signal (where as the fixation dot disappeared when the saccade signal appeared in the current study), the fixation duration varied from 300-700 ms (700-1500 ms in the current study), the response period varied from 50-400 ms (50-1500 ms in the current study), the saccade velocity threshold was 28.6°/s (50°/s in the current study), and eye position was sampled at 250 Hz (1100 Hz in the current study). Also, the reference electrode was placed on the shoulder (deltoid muscle) in Kanai, Muggleton ( 2 ) but on the upper arm in the current study. Although these design differences may have influenced the results, none of these design differences should affect performance asymmetrically, and thus they cannot explain asymmetries present in Kanai, Muggleton ( 2 ) but not in the current study. Hence, the use of basic measurements to position the electrodes seems the most likely factor underpinning the discrepant results. 

 The lack of benefits in older adults came as a particular surprise given that they have far more room for improvement and past research indicates that tDCS can confer greater benefits in older adults ( 19 ). One factor that may have contributed to the failure to induce improvements in saccadic eye movement control in the older adults pertains to age-related increases in cerebral spinal fluid ( 35 ), which can attenuate electric field strength ( 36 ). Another factor that may have reduced the chances of inducing benefits in the older adults is that the tDCS protocol used may not suit older adults due to agerelated changes in brain activation patterns ( 37 ). As reviewed in Dayan, Censor ( 38 ), small electrodes stimulate more focally, which can be beneficial in some circumstances. However, given that older adults normally show widespread prefrontal activation not seen in young adults especially when engaged in higher level cognitive processing (see 13, for a review), focal stimulation may not be optimal to induce pervasive physiological changes necessary to enhance saccadic eye movement control in older adults. 

 Another factor that may have contributed more generally to the lack of tDCS effects pertains to the spatial distribution of the induced electric field. As demonstrated in Moliadze, Antal ( 39 ), the reference electrode positioning determines the direction of current flow whilst the distance between the electrodes determines where the peak electric field is focused. Given that current passes between the two electrodes, an anode placed over the frontal region and a cathode (i.e., reference electrode) placed over the deltoid muscle or upper arm leads to the current flowing in from the anodal electrode site, passing through the brainstem and the spinal cord, and diffusing at the site of the reference electrode ( 40 ). This tDCS montage, used in the current study and in Kanai, Muggleton ( 2 ), should have resulted in the electric field concentration (i.e., the “hotspot”) being distributed outside of prefrontal regions, roughly around the neck region. Thus, the electrode positions used here and in Kanai, Muggleton ( 2 ) may not be optimal for inducing physiological changes in prefrontal regions. 

 With respect to developing a tDCS protocol that is more likely to induce physiological changes required to improve functioning, especially in older adults, future studies should take into consideration using a contralateral encephalic reference electrode (e.g., over the forehead or cheek), which should optimize the electric field in prefrontal regions ( 41 ). This arrangement, usually involving a large active electrode over prefrontal cortex combined with a contralateral encephalic reference electrode, has shown promise in a large number of studies that reported improvements in non-oculomotor cognitive functions in older adults ( 42, 43 ). This more typical montage may be worthy of assessment in relation to oculomotor functions as well. 

## Conclusions

 In conclusion, the current study found no evidence that anodal tDCS over frontal subregions improves saccadic eye movement behavior. The failure to produce benefits using a more clinically practical protocol, adapted from Kanai, Muggleton ( 2 ), suggests that localization of the FEF may be necessary for this smallelectrode tDCS protocol to be effective. Future efforts to develop a clinically practical protocol should consider using a larger active electrode and positioning the active and reference electrodes such that the maximally stimulated brain regions are relevant to the functions targeted in the population under study. In addition, a sham stimulation control condition should always be included to enable confirmation that any apparent benefits in active stimulation conditions are attributable to the tDCS. 

### Ethics and Conflict of Interest

The authors declare that the contents of the article are in agreement with the ethics described in http://biblio.unibe.ch/portale/elibrary/BOP/jemr/ethics.ht ml and that there is no conflict of interest regarding the publication of this paper.

### Acknowledgements

This research was supported by the University of Otago and the Neurological Foundation of New Zealand (grant number 1410-SPG).

## Appendix

**Table 2:**
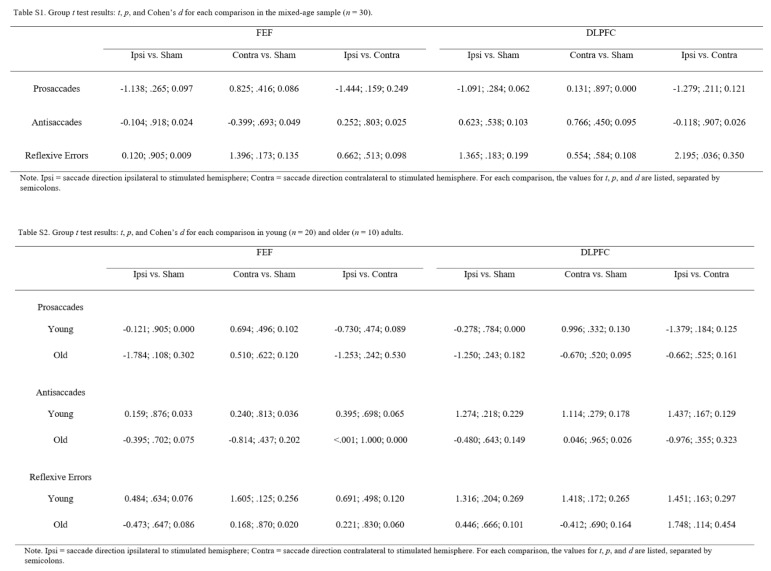
Table 2.Group t-test results: *t,p,* and *Cohen’s d* for each comparison in young (*n*=20) and older (*n*=10) adults.
